# Computational Methods to Work as First-Pass Filter in Deleterious SNP Analysis of Alkaptonuria

**DOI:** 10.1100/2012/738423

**Published:** 2012-04-19

**Authors:** R. Magesh, C. George Priya Doss

**Affiliations:** ^1^Department of Biotechnology, Faculty of Biomedical Sciences, Technology & Research, Sri Ramachandra University, Chennai 600116, India; ^2^Centre for Nanobiotechnology, Medical Biotechnology Division, School of Biosciences and Technology, VIT University, Tamil Nadu, Vellore 632014, India

## Abstract

A major challenge in the analysis of human genetic variation is to distinguish functional from nonfunctional SNPs. Discovering these functional SNPs is one of the main goals of modern genetics and genomics studies. There is a need to effectively and efficiently identify functionally important nsSNPs which may be deleterious or disease causing and to identify their molecular effects. The prediction of phenotype of nsSNPs by computational analysis may provide a good way to explore the function of nsSNPs and its relationship with susceptibility to disease. In this context, we surveyed and compared variation databases along with *in silico* prediction programs to assess the effects of deleterious functional variants on protein functions. In other respects, we attempted these methods to work as first-pass filter to identify the deleterious substitutions worth pursuing for further experimental research. In this analysis, we used the existing computational methods to explore the mutation-structure-function relationship in *HGD* gene causing alkaptonuria.

## 1. Introduction

Understanding the genomic variation in the human population is one of the primary challenges of current genomics research. Identifying genenomic variations that underlie the etiology of human diseases is of primary interest in current molecular epidemiology, medicine, and pharmarcogenomics [1]. Nevertheless, our understanding of the genetic etiology of human disease is still limited due to the enormous number of genetic variations on the human genome. Genetic variation in the human genome takes many forms, ranging from large, microscopically visible chromosome anomalies to single-nucleotide changes. The simplest form of these variations is the substitution of one single nucleotide for another, termed as “Single Nucleotide Polymorphism” (SNP). They are more common than other types of polymorphisms and the number of SNPs in each individual is said to be in the range of 3–5 million [2]. Analysis of Human Gene Mutation Database (HGMD) [3] has revealed that the vast majority of known monogenic disease cases act through changes to the coding sequence, with missense mutations (a single base change resulting in change of a single amino acid) accounting for greater than 60% of all monogenic disease mutations. SNPs can contribute directly to disease predisposition by modifying the function of a gene, or they can be used as a marker to find nearby disease causing mutations through association or family-based studies. SNPs that change the encoded amino acids are called nonsynonymous single nucleotide polymorphisms (nsSNPs); SNPs do not change the amino acids and are called synonymous SNPs. However, most SNPs occur in the intronic regions. Study of intronic SNPs is also important because of their influence on gene expression which can be occurred through different molecular pathways such as changing regulatory elements, splicing patterns, up-and downregulation of exonic splice enhancers (ESE), and intronic splice enhancers (ISE) [4]. Half of all genetic changes related to human diseases are attributed to nsSNP variants [3]. Therefore, these nsSNPs with dramatic phenotypic consequences are usually considered as “deleterious nsSNPs,” in contrast to “tolerant nsSNPs” without phenotypic changes. To differentiate deleterious nsSNPs from tolerant nsSNPs is of great importance for understanding the genetic basis of alkaptonuria, especially for clarifying individual variability to* HGD* deficiency in human.

Because most sequence variants are SNPs, a massive effort has been undertaken by several private and public organizations [5, 6] and opens the way for the development of a detailed understanding of the mechanisms by which genetic variation results in phenotype variation. Currently, most of the diseases represented by the genes in the databases like OMIM [7], HGMD [3], and Swiss-Prot [8] segregate in a Mendelian manner, which suggests that they are caused by single deleterious lesions. Computational tools like SIFT [9] and PolyPhen [10] are able to predict 90% of damaging SNPs. Over the past few years, there have been many computational methods developed to predict whether a missense mutation is deleterious to the structure or the function of the gene and will therefore lead to disease based on sequence- and/or structure-based methods [9–14]. These methods classify the mutations into whether they have negative, neutral, or positive effects on the structure or function of the proteins. Predictions regarding missense mutations can be supported by comparative evolutionary analysis to establish whether mutations are situated in conserved genomic regions. These prediction methods can help us to narrow down candidate nsSNPs to identify the causative lesion within a large genomic region implicated in disease by linkage studies [15]. In this inquest, we employed two diverse approaches in computational analysis of deleterious nsSNPs, namely, empirical rule-based method and support vector method. SIFT and PolyPhen are based on empirical rules while I-Mutant 2.0 [16] is based on support vector method. Knowledge of the three-dimensional structure of a gene product is of major assistance in predicting and understanding its function, its role within the cell, and its role in disease. Proteins with mutations do not always have 3D structures that are solved and deposited in PDB. Therefore, it is necessary to construct 3D models by locating the mutation in 3D. This is a simple way of detecting what kind of adverse effects that a mutation can have on a protein. To analyze the correlation between structure and mutation, we have analysed the mutant 3D structures by solvent accessibility, secondary structure analysis, and the change of stability affected by mutation. Deleterious missense mutations analyses for the *HGD *gene causing alkaptonuria have not been estimated computationally till now by modelling approach, although they have received great focus from experimental researchers. To answer this question, in the absence of further experimental investigations, we tested different models of programs, to discriminate deleterious missense mutations from neutral ones in alkaptonuria.

Alkaptonuria (MIM # 203500) is a rare autosomal recessive disorder of the phenylalanine and tyrosine catabolic pathway caused by the deficiency of homogentisate dioxygenase (HGO, EC 1.13.11.5). AKU was the first disease to be interpreted as a single gene trait and the mode of inheritance was reported by 2002 Garrod and Oxon [17]. This enzyme deficiency results in the accumulation of homogentisic acid (HGA), an intermediary metabolite in phenylalanine and tyrosine catabolism. Biochemical evidence of the defect in AKU was provided by La Du et al. in 1958, exactly fifty years later. He demonstrated the absence of HGO activity in a liver homogenate prepared from an AKU patient and established that the defect was limited to HGO, suggesting that in affected individuals there is a failure to synthesize active enzyme [18]. The clinical manifestation of this disease is urine that turns dark on standing and alkalinization, black ochronotic pigmentation of cartilage and collagenous tissues, and arthritis, especially characteristic in the spine. The gene responsible for AKU was located in human to 3q21–q23 [19]. *HGD *contains 14 exons and covers 60 kb of genomic DNA [19]. It encodes a 49,973 dalton, 445 amino acid protein that forms a dimer of two trimers giving rise to a functional hexamer. The crystalline structure of the *HGD* protein has been resolved [20]. AKU presents a remarkable allelic heterogeneity. More than 40 different AKU mutations have been reported till date [21].

## 2. Materials and Methods

### 2.1. Evaluation of Dataset

The SNPs information (Protein accession number (NP), mRNA accession number (NM) and SNP ID) of *HGD* gene was retrieved from the NCBI (http://www.ncbi.nlm.nih.gov/projects/SNP/) [6], and SWISS-Prot database (http://ca.expasy.org/sprot/) [8] for our analysis. We examined three different annotation categories: (a) synonymous/nonsynonymous (b) splicing, and (c) regulatory SNPs. Nonsynonymous SNPs occur in the coding region of the genome and affect whether or not the amino acid is changed. Splicing SNPs occur at the intron/exon boundary when proteins are being made; an SNP at this location can affect how the intron is spliced out. Regulatory SNPs occur in the promoter of the sequence and are thought to affect gene expression. The data on human *HGD* gene was collected from Online Mendelian Inheritance in Man (OMIM) http://www.ncbi.nlm.nih.gov/omim [7] and Entrez Gene on NCBI Web site http://www.ncbi.nlm.nih.gov/Genbank/ [6]. The information on the correlation between the nsSNPs and alkaptonuria disease was compiled from *in vivo* and *in vitro* experiments according to PubMed  (http://www.ncbi.nlm.nih.gov/PubMed/),  OMIM (http://www.ncbi.nlm.nih.gov/omim/), and UniProtKB/Swiss-Prot databases (http://ca.expasy.org/sprot/).

### 2.2. I-Mutant 2.0

Over the past few years, there have been many computational methods utilizing machine-learning techniques (support vector machines, neural networks, and decision trees) that have been applied successfully in sequence-structure relationships predictions. Support vector machines (SVMs) are universal classifiers that learn a variety of data distributions from training samples and, as such, are applicable to classification and regression tasks [22]. We applied I-Mutant 2.0, a support vector machine- (SVM-) based tool for the automatic prediction of protein stability. I-Mutant 2.0 predictions are performed starting either from the protein structure or, more importantly, from the protein sequence [16]. This program was trained and tested on a data set derived from ProTherm [23], which is presently the most comprehensive available database of thermodynamic experimental data of free energy changes of protein stability upon mutation under different conditions. The output file shows the predicted free energy change value or sign (DDG) which is calculated from the unfolding Gibbs free energy value of the mutated protein minus the unfolding Gibbs free energy value of the native type (kcal/mol). If the DDG value is positive, then the mutated protein will have high stability and vice versa for less stability [16].

### 2.3. SIFT

Sorting intolerant from tolerant (SIFT) software developed by Kumar et al. [9] predicts whether an amino acid substitution affects protein function based on sequence homology and the physical properties of amino acids. SIFT uses sequence homology among related genes and domains across species to predict the impact of all 20 possible amino acids at a given position, allowing users to determine which nsSNPs would be of most interest to study by sorting variants by this prediction score. It takes a query sequence and uses multiple alignment information to predict tolerated and deleterious substitutions for every position of the query sequence. The underlying principle of this program is that it generates alignments with a large number of homologous sequences and assigns scores to each residue, ranging from zero to one. Scores close to zero indicate evolutionary conservation and intolerance to substitution, while scores close to one indicate tolerance to substitution. The SIFT prediction is given as a tolerance index (TI) score ranging from 0.0 to 1.0, which is the normalized probability that the amino acid change is tolerated. SIFT scores less than or equal 0.05 are predicted by the algorithm to be intolerant or deleterious amino acid substitutions, whereas scores greater than 0.05 are considered tolerant. SIFT scores were classified as intolerant (0.00–0.05), potentially intolerant (0.051–0.10), borderline (0.101–0.20), or tolerant (0.201–1.00) [24]. The higher the tolerance index of a particular amino acid substitution is, the lesser is its likely impact.

### 2.4. PANTHER

PANTHER version 7 (Protein Analysis Through Evolutionary Relationships) estimates the likelihood of a particular nsSNP to cause a functional impact on the protein [25]. It calculates the subPSEC (substitution position-specific evolutionary conservation) score based on an alignment of evolutionarily related proteins. The algorithm takes the absolute value in order to make the scores symmetric and then multiplies by −1 to adhere to the substitution matrix convention that more negative scores correspond to more severe substitutions. When subPSEC = 0, the substitution is interpreted as functionally neutral, whereas more negative values of subPSEC predict more deleterious substitutions. The cutoff subPSEC −3 indicates a deleterious substitution [26].

### 2.5. PolyPhen

PolyPhen differs from SIFT in that it predicts how damaging a particular variant may be by using a set of empirical rules based on sequence, evolutionary conservation, and structural information characterizing a particular variant. PolyPhen is a multiple sequence alignment server that aligns sequences using structural information. Input for the PolyPhen server is either a protein sequence or a SWALL database ID or accession number together with sequence position with two amino acid variants. We submitted the query in the form of sequence with mutational positions each with two amino acid variants. In addition to using sequence alignments, PolyPhen utilizes protein structure databases, such as PDB (Protein Data Bank) or PQS (Protein Quarternary Structure), DSSP (Dictionary of Secondary Structure in Proteins), and three-dimensional structure databases to determine if a variant may have an effect on the protein's secondary structure, interchain contacts, functional sites, and binding sites [10]. Then, it calculates position-specific independent counts (PSICs) scores for each of two variants and computes the difference of the PSIC scores of the two variants. The higher a PSIC score difference is, the higher is the functional impact a particular amino acid substitution is likely to have. A PSIC score difference of 1.5 and above is considered to be damaging. PolyPhen scores were designated probably damaging (≥2.00), possibly damaging (1.50–1.99), potentially damaging (1.25–1.49), borderline (1.00–1.24), or benign (0.00–0.99) according to the classification proposed by Xi et al. [27].

### 2.6. FASTSNP

In order to efficiently identify nsSNPs with a high possibility of having a functional effect, FASTSNP tool was applied for the detection of nsSNP influence on cellular and molecular biological function, for example, transcriptional and splicing regulation. The online tool FASTSNP [28] (http://fastsnp.ibms.sinica.edu.tw/pages/input_CandidateGeneSearch.jsp) was used for predicting the functional significance of the nsSNPs, 3′ and 5′ UTR SNPs and also to identify the polymorphism involving intron which may lead to defects in mRNA processing. The FASTSNP follows the decision tree principle with external web service access to TFSearch, which predicts whether a noncoding SNP alters the transcription factor binding site of a gene. The score is given on the basis of levels of risk with a ranking of 0, 1, 2, 3, 4, or 5. This signifies the levels of no, very low, low, medium, high, and very high effect, respectively.

### 2.7. Structural Analysis

Structural analyses were performed based on the crystal structure of the protein for evaluating the structural stability of native and mutant protein. We used the SAAPdb [29] and dbSNP to identify the protein coded by *HGD* gene in chain “A” of PDB ID 1EY2 [30]. We also confirmed the mutation residues and their position from this server. These mutation residues and their corresponding positions were in complete agreement with the results obtained with SIFT and PolyPhen programs. The mutation analysis was performed in the “A” chain of the 1EY2 using SWISSPDB viewer [31], and energy minimization for three-dimensional structures was performed using NOMAD-Ref server [32]. This server use Gromacs as default force field for energy minimization based on the methods of steepest descent, conjugate gradient and L-BFGS methods [33]. We used the conjugate gradient method for optimizing the three-dimensional structures. Computing the energy gives the information about the protein structure stability. Deviation between the two structures was evaluated by their RMSD values.

### 2.8. Analyzing the Effects of Mutations on Protein Stability

The structure and function of proteins are determined by various factors. To check the stability of the native and mutant modeled structures, identification of the stabilizing residues is useful. We used the server SRide [34] for identifying the stabilizing residues in native protein and in the mutant model. Stabilizing amino acids can be predicted based on long-range interactions in protein structures and hydrophobicity and conservation of amino acid residues. Mutations found at stability centers were considered by us to be destabilizing and thus deleterious. SRide combines several methods to identify residues expected to play key roles in stabilization. It analyzes tertiary structures, rather than primary structures, and the evolutionary conserved residues contained within. A residue is predicted to be stabilizing if it is surrounded by hydrophobic residues, exhibits long-range order, has a high conservation score, and is part of a stability center.

## 3. Results and Discussion

### 3.1. Predictions of Deleterious nsSNPs in the Coding Region of HGD Gene

The functional impact of nsSNPs can be assessed by evaluating the importance of the amino acids they affect. We employed four widely used computational tools for determining the functional significance of nsSNPs. In this analysis, we applied two different approaches in computational analysis of deleterious nsSNPs, namely, empirical rule-based method and Support Vector Method (SVM). These approaches use alternative classification methods to decide which of the nsSNPs may have deleterious or neutral phenotypes. SVM approaches, a set of trained data, and trained attributes are required to forecast precisely the effects of amino acid substitutions on various protein properties such as protein stability, protein secondary structures, solvent accessibility of residues, residue-residue interactions, and protein 3D structures [38]. Both these different methods use sequence information, structural information, or both. Sequence- (SIFT, PANTHER) and structure-based methods (PolyPhen, I-Mutant 2.0) are the most common approaches used in SNP prediction tools. Structure-based approach is not feasible to implement for the proteins with unknown 3D structures. Hence, sequence-based prediction methods have more advantage over the structure-based ones, as they include all types of effect at the protein level and can be applied to any human protein with known relatives. Tools that integrate both sequence and structure information have the added advantage of being able to assess the reliability of the generated prediction results by cross-referencing the results from both approaches. Tools that combine these approaches (PolyPhen and I-Mutant 2.0) use different algorithms and methodologies for prediction, thereby having a wider coverage of the different aspects of SNP analysis. Both these methods have disadvantages and advantages in predicting the effects of SNPs on protein stability. The user must decide which tool is most suited to the specific objectives of their analysis to gain the optimum knowledge. Although the predictive power of protein structural information has been established, a comparison between structure-based and sequence-based methods is still needed in monogenic diseases. In this pipeline, we analysed deleterious substitutions of *HGD *which are accountable for alkaptonuria (AKU) is the first disease to be interpreted as a single gene trait. The SIFT [9] and PolyPhen [16] are the representatives for empirical rules-based method. They make predictions based on knowledge of the functional sites of the protein, positional residue variation in sequence alignments, and the 3D structure of the protein. The results are outlined in Table 1. The SIFT was used to determine the conservation level of a particular amino acid position in a protein, which leads to a tolerance index score ranging from 0.0 to 1.0 for SNP functionality. The protein sequences of 22 nsSNPs were submitted independently to the SIFT program to check its tolerance index. The SIFT algorithm deploys sequence homology to calculate a score, determining the evolutionary conservation status of the amino acid of interest and predicting whether its substitution will affect protein function. Substitutions at specific positions showing normalized probabilities less than the chosen cutoff value of 0.05 are predicted to be deleterious, and those greater than or equal to 0.05 are predicted to be tolerated. We identified a total of 11 of 22 nsSNPs that were scored as intolerant by SIFT scores of 0.0. The PANTHER software calculates SubPSEC scores—substitution position-specific evolutionary conservation. A SubPSEC score of −3 or less means that the substitution has probable functional implications. Out of the 22 nsSNPs submitted to PANTHER, eight nsSNPs were found to be deleterious and exhibited subPSEC score range of −3.08256 to −5.98264. From Table 1 it can be seen that G161R has the lowest score (−5.98264), indicating a strong probability of functional impact. PANTHER identified all the substitutions to be deleterious as same as SIFT, except D326N, M368V, T369N, E379Q, and P373L which are found to be having SubPSEC score of −2.1555, −2.45276, −2.88444, −2.62114, and −2.72749, respectively. Predictions of how a particular nsSNP may affect protein structure by PolyPhen 2.0 are assigned as “probably damaging,” a score made with high confidence that the nsSNP should affect protein structure and/or function; “possibly damaging,” where it may affect protein function and/or structure; and “benign,” as most likely having no phenotypic effect. PolyPhen identified a total of 12 of 22 nsSNPs that were scored as damaging and exhibited a PolyPhen score of more than 1.5. All the 22 nsSNPs submitted to SIFT, PANTHER, and PolyPhen were also submitted as input to the I-Mutant 2.0 server. The more negative the DDG value is, The less stable the given point mutation likely to be, as predicted by I-Mutant 2.0 server. The more the negative DDG value is, The less stable is the given point mutation is likely to be as predicted by I-Mutant 2.0 server. Among the 22 nsSNPs, 16 were found to be less stable and exhibited a DDG value ranging from −0.35 to −5.21, respectively. The nsSNPs which were predicted to be deleterious in causing an effect in the structure and function of the protein by SIFT, PANTHER, PolyPhen, and I-Mutant2.0 correlated well with experimental studies as shown in Table 1 [35, 39, 40]. By comparing the scores of all the four methods used in this analysis, 7 nsSNPs with IDs rs28941783, rs28942100, rs120074174, rs120074170, rs120074171, rs139501220, and rs120074172 were predicted to be functionally significant

### 3.2. Predictions of Deleterious nsSNPs and UTR SNPs

The functional prediction of SNPs in untranslated region for the *HGD* gene has not been estimated computationally until now, although they have been the focus for experimental researchers. Therefore in this work, we used FASTSNP for this analysis. FASTSNP tool helps in classifying and prioritizing phenotypic risks and deleterious effects of SNPs based upon their influence over determining protein structure, pre-mRNA*∖*splicing, deviation in transcriptional levels of the sequence, alterations in the premature translation termination, deviations in the sites at promoter region for transcription factor binding, and so forth. By FASTSNP, seven nsSNPs and two UTR SNPs were found to be functionally significant. Out of which 2 SNPs were predicted to affect the splicing site with a risk ranking of 3-4, 5 SNPs were predicted to affect splicing regulation with a risk ranking of 2-3, and 2 SNPs were predicted to affect Promoter/regulatory region with a risk ranking of 1–3 respectively (Table 2). Further, we extended our analysis by comparing FASTSNP with SIFT, PolyPhen, I-Mutant 2.0, and PANTHER. NsSNPs with an ID rs28942100, and rs28941783 predicted by FASTSNP, were found to be deleterious by SIFT/PolyPhen/PANTHER/I-Mutant 2.0 highlighted as bold in Table 2.

### 3.3. Modelling of Deleterious nsSNPs in HGD Gene

**Figure 1:**
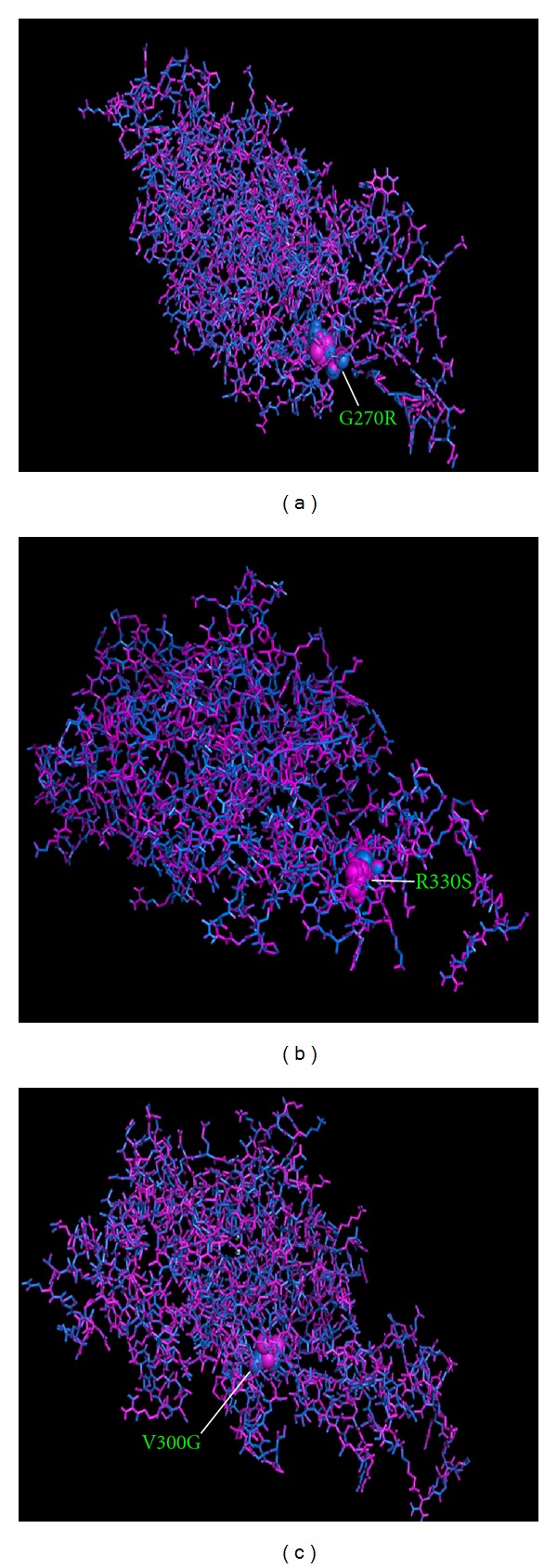
Superimposed structures of native and mutant modeled of *HGD* gene were visualized in stick model using PyMOL release 0.99. (a) Superimposed structure of native amino acid Glycine in sphere shape (blue color) with mutant amino acid Argenine (violet) at position 270 in PDB ID 1EY2 (chain A) of *HGD* gene with RMSD 1.38 Å. (b) Superimposed structure of native amino acid Argenine in sphere shape (blue color) with mutant amino acid Serine (violet) at position 330 in PDB ID 1EY2 (chain A) of *HGD* gene with RMSD 1.21 Å. (c) Superimposed structure of native amino acid Valine in sphere shape (blue color) with mutant amino acid Glycine (violet) at position 300 in PDB ID 1EY2 (chain A) of *HGD* gene with RMSD 1.10 Å.

Knowledge of the 3D structure of a gene product is of major assistance in understanding the function within the cell and its role in causing disease. Proteins with mutations do not always have 3D structures that are analyzed and deposited in Protein data bank (PDB). Therefore, it is necessary to construct 3D models by locating the mutation in 3D structures. This is a simple way of detecting what kind of adverse effects that a mutation can have on a protein. The linear sequence of amino acids specifies the 3D structure of the protein. Even as single amino acid substitution can cause a disruption in structure of a protein by affecting its stability, this leads to change in structural and thermodynamic properties affecting the protein dynamics. Mutation analysis was performed based on the results obtained from highest SIFT, PolyPhen, I-Mutant 2.0, and PANTHER scores. The mutations at their corresponding positions were performed by SWISS-PDB viewer independently to achieve modelled structures. Then, energy minimizations were performed by NOMAD-Ref server for the native type protein and mutant type structures. According to this in *HGD* gene, mutation occurred for native protein in “A” of PDB ID 1EY2 at position G161R with SNP ID rs28941783, P230S with SNP ID rs28942100, G270R with SNP ID rs120074174, V300G with SNP ID rs120074170, R330S with SNP ID rs120074171, M339I with SNP ID rs139501220, and, H371R with SNP ID rs120074172. Computing the energy minimization gives the information about the protein structure stability. We checked the total energy for mutant type structure G161R, P230S, G270R, V300G, R330S, M339I, and H371R that were found to be −23429.56 Kcal/mol, −23486.42 Kcal/mol, −23528.21 Kcal/mol, −23869.20 Kcal/mol, −23398.63 Kcal/mol, −23489.12 Kcal/mol, and −23528.53 Kcal/mol. Divergence in mutant structure with native structure is due to mutation, deletions, and insertions [36], and the deviation between the two structures is evaluated by their RMSD (root mean square deviation) values which could affect stability and functional activity [37]. The higher the RMSD value is, the more will be the deviation between native and mutant type structures and which in turn changes their functional activity. The RMSD values between the native type (1EY2) and the mutant type structure G161R, P230S, G270R, V300G, R330S, M339I, and H371R were found to be 1.34 Å, 1.20 Å, 1.38 Å, 1.10 Å, 1.21 Å, 1.24 Å, and 1.36 Å. The RMSD values of all mutant structures were found to be similar. The higher the RMSD value is, the more will be the deviation between native and mutant type structures and which in turn changes their functional activity. G161R, P230S, and G270R involve change in polarity from nonpolar amino acid to polar amino acid which can cause major differences in proteins that can subvert their normal functions. The V300G involves change from valine to glycine, both being *nonpolar* amino acids, with reduction in value of hydropathy index from 4.2 to −0.4 while M339I involves change from methionine to Isoleucine with increase in value of hydropathy index from 1.9 to 4.5, thus affecting the hydrophobic interactions. The R330S involves change from argentine to serine, and H371R involves change from histidine to argentine, both being polar amino acids, respectively. The mutational exchange of a polar amino acid for a *nonpolar* one and vice versa must be more dangerous than the transition from one polar group to another or from a *nonpolar* to another *nonpolar* one [41]. Among the six deleterious nsSNPs predicted by the four computational methods, G161R, P230S, and, G270R must be considered highly deleterious substitution based on the transition in polarity from *nonpolar* to polar group. The superimposed structures of the native protein 1EY2 (chain A) with the mutant type proteins G270R, R330S, and V300G *HGD* gene are shown in (Figures 1(a), 1(b), and 1(c)). These figures were drawn using PyMOL release 0.99 [42]. We further extended our analysis by using SRide tool for identifying the stabilizing residues which plays an important role in stabilization of protein. We analysed native and mutant proteins (G161R, P230S, G270R, V300G, R330S, M339I, and H371R) of *HGD* gene. From this analysis fifteen stabilising residues, namely, LEU61, TYR62, THR118, HIS134, PHE136, ASN139, PRO157, ARG194, GLY195, VAL200, VAL262, VAL263, ALA264, TRP265, and VAL312 were found to be common in both native structure (1EY2) and mutant model G161R, P230S, G270R, V300G, R330S, and M339I of *HGD* gene. Most importantly stabilising residue VAL312 was missing in the mutant model H371R. Based on the RMSD value, total energy, polarity, and SRide, we predict that these nsSNPs (G161R, P230S, G270R, V300G, R330S, M339I, and H371R) may lead to decrease in stability of the protein and cause alkaptonuria. These results will definitely provide base for the in-depth research into the effect of these six nsSNPs onto the structure as well as influence of altered response to drug response, susceptibility of the disease, and phenotypic variations.

## 4. Conclusion

In addition to the molecular approaches, which are laborious and time-consuming, it is now possible to apply computational approaches to filter out deleterious substitutions that are unlikely to affect protein function. Alternatively, computational approaches, which are fast and relatively inexpensive methods, can offer a more feasible means for phenotype prediction based on the biochemical severity of the amino acid substitution and the protein sequence and structural information. Computational analysis performed here suggests that individual tools correlate modestly with observed results and by combining information from a variety of tools may significantly increase the predictive power for determining the functional impact of a given SNP. Different computational methods employed in this analysis have its own advantages and disadvantages in predicting the functional SNPs. The user must decide which tool is most suited to the specific objectives of their analysis to gain the optimum knowledge. This SNP prioritization analysis integrates relevant biomedical information and computational methods to provide a systematic analysis of functional and deleterious nsSNPs. In other respects, we attempted these methods to work as first-pass filter to identify the deleterious substitutions worth pursuing for further experimental research.

## Figures and Tables

**Table 1:** List of nsSNPs predicted to be deleterious by SIFT, PolyPhen, PANTHER, and I-Mutant 2.0 in the coding region of *HGD *gene.

rs IDs	Allele frequency and change	AA position	SIFT		PolyPhen		PANTHER		I-Mutant 2.0		Reference
			Tolerance index	Predicted impact	PSIC score	Predicted impact	subPSEC score	Predicted impact	DDG	Predicted impact	
rs138356501	A(0.000)/T(1.000)	Y37F	0.15	Tolerant	0.534	Benign	−1.92527	Tolerated	0.01	Increase stability	
rs138846036	A(0.012)/C(0.988)	A48S	0.12	Tolerant	0.497	Benign	−2.05903	Tolerated	−0.59	Decrease stability	
rs141965690	A(0.000)/T(1.000)	E74V	0.29	Tolerant	0.524	Benign	−2.29059	Tolerated	0.33	Increase stability	
rs2255543	A(0.262)/T(0.738)	Q80H	0.45	Tolerant	0.258	Benign	−1.49933	Tolerated	−1.17	Decrease Stability	[35]
rs35702995	A(0.996)/C(0.004)	E87A	0.50	Tolerant	0.881	Benign	−2.18204	Tolerated	−1.85	Decrease Stability	
rs143267384	A(0.000)/T(1.000)	E101V	0.06	Tolerant	1.817	Probably damaging	−2.67878	Tolerated	0.82	Increase stability	
**rs28941783**	A(0.000)/G(1.000)	**G161R**	**0.00**	Intolerant	**2.711**	Probably damaging	**−5.98264**	Deleterious	**−2.23**	Decrease Stability	[36]
rs140543217	A(0.000)/G(1.000)	L163F	0.00	Intolerant	1.105	Benign	−3.70687	Deleterious	−1.12	Decrease stability	
**rs28942100**	C/T (No frequency)	**P230S**	**0.00**	Intolerant	**2.986**	Probably damaging	**−5.30874**	Deleterious	**−1.72**	Decrease Stability	[35, 36]
**rs120074174**	A(0.000)/G(1.000)	**G270R**	**0.00**	Intolerant	**2.790**	Probably damaging	**−5.85971**	Deleterious	**−0.40**	Decrease Stability	[36]
rs148641817	G(1.000)/T(0.000)	A293E	0.09	Tolerant	1.128	Benign	−1.96145	Tolerated	0.84	Increase stability	
**rs120074170**	G/T (No frequency)	**V300G**	**0.00**	Intolerant	**2.975**	Probably damaging	**−4.393**	Deleterious	**−5.21**	Decrease Stability	[35, 36]
rs143556739	A(0.001)/G(0.999)	R307C	0.01	Intolerant	1.535	Probably damaging	−3.80886	Deleterious	−1.59	Decrease stability	
rs143396290	C(1.000)/T(0.000)	D326N	0.02	Intolerant	0.503	Benign	−2.1555	Tolerated	0.37	Increase stability	
**rs120074171**	G/T (No frequency)	**R330S**	**0.00**	Intolerant	**2.830**	Probably damaging	**−3.2862**	Deleterious	**−3.53**	Decrease Stability	[37]
**rs139501220**	A(0.000)/C(1.000)	**M339I**	**0.00**	Intolerant	**2.858**	Probably damaging	**−3.23432**	Deleterious	**−2.26**	Decrease stability	
rs120074173	A(1.000)/G(0.000)	M368V	0.00	Intolerant	2.373	Probably damaging	−2.45276	Tolerated	−0.35	Decrease Stability	[37]
rs149326001	G(1.000)/T(0.000)	T369N	0.00	Intolerant	1.535	Probably damaging	−2.88444	Tolerated	−0.60	Decrease stability	
**rs120074172**	A/G (No frequency)	**H371R**	**0.00**	Intolerant	**3.419**	Probably damaging	**−3.08256**	Deleterious	**−0.95**	Decrease Stability	[37]
rs150145204	C(0.001)/G(0.999)	D376E	0.84	Tolerant	0.089	Benign	−1.09282	Tolerated	0.14	Increase stability	
rs141753513	C(1.000)/G(0.000)	E379Q	0.01	Intolerant	1.096	Benign	−2.62114	Tolerated	−0.38	Decrease stability	
rs138558042	A(0.000)/G(1.000)	P373L	0.00	Intolerant	2.074	Probably damaging	−2.72749	Tolerated	−0.66	Decrease stability	

Highly deleterious by SIFT, Panther, PolyPhen and I-Mutant were indicated as bold.

**Table 2:** List of SNPs that were predicted to be functional significance by FASTSNP.

SNPs ID	Allele frequency and change	Region	Possible functional effect	Ranking and Level of risk
rs7652072	A/G (No frequency)	Intron	Splicing site	3-4 (Medium to high)
rs55661952	C/T (No frequency)	5′UTR (−201A>G)	Promoter/regulatory region	1–3 (Low to medium)
rs2733829	C/T (No frequency)	5′UTR (−339C>T)	Promoter/regulatory region	1–3 (Low to medium)
**rs28942100**	C/T (No frequency)	nsSNP **(P230S)**	Missense (conservative)	2-3 (Low to medium)
**rs28941783**	A(0.000)/G(1.000)	nsSNP **(G161R)**	Missense (conservative); Splicing regulation	2-3 (Low to medium)
rs35702995	A(0.996)/C(0.004)	nsSNP (E87A**) **	Missense (conservative); Splicing regulation	2-3 (Low to medium)
rs2255543	A(0.514)/T(0.700)	nsSNP (Q80H)	Missense (conservative); Splicing regulation	2-3 (Low to medium)
rs2293734	G/T (No frequency)	csSNP (P158P**)**	Sense/synonymous; Splicing regulation	2-3 (Low to medium)

SNP IDs which were highlighted in bold were found to be deleterious by SIFT, PANTHER, PolyPhen and I-Mutant 2.0.
